# Laser Boronizing of Additively Manufactured 18Ni-300 Maraging Steel Part Surface

**DOI:** 10.3390/ma15134631

**Published:** 2022-07-01

**Authors:** Jelena Škamat, Kęstutis Bučelis, Olegas Černašėjus

**Affiliations:** 1 Laboratory of Composite Materials, Vilnius Gediminas Technical University, 08217 Vilnius, Lithuania; 2 Department of Mechanical and Materials Engineering, Vilnius Gediminas Technical University, 08217 Vilnius, Lithuania; kestutis.bucelis@vilniustech.lt (K.B.); olegas.cernasejus@vilniustech.lt (O.Č.)

**Keywords:** maraging steel, laser boronizing, selective laser melting, hardness, microstructure, XPS

## Abstract

The problem of insufficient wear resistance of maraging steels (MSt) has so far been solved mainly by the use of the thermochemical nitriding process, which has a number of limitations and disadvantages. In the present work, for MSt parts manufactured by laser powder bed fusion (LPBF), a more flexible laser alloying process was suggested as an alternative surface hardening process. The purpose of the present work is to give a better understanding on the possible hardening effect obtainable when amorphous boron is used as an alloying additive in relation with microstructural evolution and specific process parameters and to promote further development of this technology. For the alloying, a one kilowatt CO_2_ laser was applied at 0.5–4.0 mm laser spot and 250–1500 mm/min laser operating speed, providing 50,955–796 W∙cm^−2^ power density and 24.0–4.0 J∙mm^−1^ heat input. Before laser processing, surfaces were covered with amorphous boron. The appropriate melt pool geometry was obtained at 0.5 mm laser spot, for which XPS analysis revealed an increase in boron concentration from ~3.1 to ~5.7 wt.% with a laser speed increase from 500 to 1500 mm/min. XRD analysis revealed domination of Fe_3_B type borides along with the presence of FeB, Fe_2_B, Ni_4_B_3_ borides, austenitic and martensitic phases. The microstructure of modified layers exhibited evolution from hypoeutectic microstructure, having ~630–780 HK0.5 hardness, to superfine lamellar nanoeutectic (~1000–1030 HK0.2) and further to submicron-sized dendritic boride structure (~1770 HK0.2). Aging of laser-boronized layers resulted in the change of phase composition and microstructure, which is mainly expressed in a plenty precipitation of Mo_2_B_5_ borides and leads to a reduction in hardness—more significant (by ~200–300 HK0.2) for hypoeutectic and hypereutectic layers and insignificant (by ~50 HK0.2) for near-eutectic. With the application of the laser boronizing technique, the hardness of MSt parts surface was increased up to ~three times before aging and up to ~2.3 times after aging, as compared with the hardness of aged MST part.

## 1. Introduction

Maraging steels (MSt) are known as a special class of high-strength steels with the specific alloying system providing highly alloyed low-carbon iron-nickel lath martensite matrix, which is aged (annealed) to obtain hardening by intermetallic precipitation. The microstructure obtained after aging provides superior properties of MSt: high yield strength combined with high fracture toughness and ease of fabrication [1,2]. Due to the lack of carbon, MSt have excellent weldability and high resistance to thermal fatigue, that makes them suitable for laser powder bed fusion (LPBF) process, which in some sources is also called selective laser melting (SLM) [3,4,5,6,7,8,9,10]. The results reported in the field showed that manufacturing of maraging steel parts by LPBF allows mechanical properties to be obtained that are of the same level as those obtained for typical wrought products [4,5,6,7,8,9,10]. The quality of sintered products—both the solid parts and lattice structures—and their properties, including fatigue limit, are largely dependent on the careful optimization of process parameters, among which the laser absorptivity is of high importance [11,12,13].

The hardening of maraging steels results from the precipitation of intermetallides in a rather soft martensite matrix (~30 HRC) [2]. The major hardener is molybdenum, which, at the first stage of aging, forms metastable Ni_3_Mo transforming into equilibrium Fe_2_Mo with further aging. At the presence of titanium and aluminum, other phases may be formed as well, such as Ni_3_Ti, Ni_3_ (Ti, Mo), Ni_3_Al, Ni_3_ (Ti, Al), (Ni, Fe)_3_ (Ti, Mo), (Fe, Ni, Co)_2_ (Mo, Ti), etc. [2,4,14,15,16]. The yield strength of MSt increases significantly (up to ~2420 MPa for commercial grades) due to the precipitation of dispersive intermetallides; however, the hardness of intermetallic compounds is not extremely high, as compared with borides, carbides, some oxides; therefore, maraging steels have moderate hardness (~58 HRC max.) and, as a result, insufficient wear resistance under severe working conditions.

It was found already in the 1960s that gas nitriding is a suitable process enabling a considerable surface hardening (up to 70 HRC or 900 HV, according to different sources) of maraging steel [17,18,19,20,21], which bases on the formation of iron nitrides. The typical steel nitriding temperatures (~450–500 °C) are close to those required for the aging of maraging steels; therefore, both the nitriding and the aging processes can be accomplished simultaneously [2]. In a more recent publication, a plasma nitriding method alone [22], as well as being followed by coating with a TiN layer by physical vapor deposition (PVD), [23] was successfully applied to harden the surface of maraging steel. One of the most significant disadvantages of thermochemical treatment is its long duration—for example, after 24 to 48 h of treatment, a ~150 μm hardened layer is typically obtained by gas nitriding [2] and a 115 μm layer was obtained with plasma nitriding after six hours of treatment in [23]. Other thermochemical processes, such as carburizing and boriding, involve the keeping of parts at high temperatures, which results in the formation of reverted austenite and a loss in strength properties [2,7]. Thermochemical processes require the use of specific equipment/cameras as well.

In the present work, a laser alloying process is suggested as an alternative to the thermochemical one. Laser surface alloying is easily controllable, universal in terms of regimes and alloying elements, typically provides rapid heating and cooling, and prevents significant thermal impact on the parent metal. Rapid development of laser manufacturing during the last decades made them cheaper and more accessible for a wider range of producers in various industries [24]. For the products of additive manufacturing by LPBF process, the introduction of surface processing technology based on the application of laser irradiation would be less complicated, both technically and in regard to personnel qualification, as compared with other processes of surface treatment. Laser alloying seems to be a more reasonable alternative, especially when local surface hardening is required.

Surface laser alloying of metals is a process that involves surface melting in the presence of one or more alloying elements or compounds, mostly, in a solid state. Due to the mixing of base metal melt with an alloying component, the surface layer with modified composition and properties is obtained. To obtain the hardening effect, hard compounds (oxides, carbides, nitrides, borides) are typically used as alloying components, or components that in reaction with metals are able to form hard intermetallides or ceramic compounds. Thus, it was shown in [25] that the laser surface alloying of AISI 1020 steel with TiN allows a uniform TiN-containing alloyed layer to be obtained, thus reducing the wear rate. Due to the formation of TiB_2_ and Ti-B-N phases, hard (1217–3351 HV0.2) coatings were obtained by the laser alloying of Ti6Al4V part with preplaced TiB_2_ and amorphous boron [26]. After the laser alloying of the copper surface with NiTi powder, the interfacial contact resistance was increased up to 7.2 times, as compared with pure copper [27]. A high hardness layer (1200 HV) was obtained on austenitic stainless steel by laser melting of the surface covered with Ti foil (5 μm) and a transparent adhesive tape (as carbon supplier), resulting in the formation of titanium carbide particles in the processed surface [28]. In the present work, amorphous boron was used as an alloying component. The conventional thermochemical boriding (or boronising) is known as the most effective method for increasing the hardness and improving the wear resistance of steel. The method is based on the equilibrium Fe-B phase diagram [29], according to which hard iron borides are forming–Fe_2_B containing 8.48 wt.% B and FeB containing 16.25 wt.% B. The hardness of FeB and Fe_2_B is 17,000–22,000 and 14,000–18,000 MPa, respectively, where lower limits correspond to the hardness of pure borides [30]. Fe_3_B boride is considered as a product of metastable solidification and is not shown in equilibrium phase diagram [31]. Thermochemically boronised surfaces typically present dual phase FeB + Fe_2_B or single phase Fe_2_B layers. In the latter case, less brittle diffusion coatings are obtained. The successful laser boronising of steels is also reported in a number of works [32,33,34,35]. Thus, the laser processing of 41Cr4 steel resulted in the formation of a hardened surface containing stable Fe_2_B and metastable Fe_3_B iron borides and martensite having 1100–1600 HV hardness in the melted zone [32]. Laser boriding of 100CrMnSi6-4 bearing steel allowed the wear resistant surface of high hardness (up to ~1450 HV) to be obtained; the presence of FeB, Fe_2_B and Fe_3_B borides along with martensite and borocementite Fe_3_ (C,B) was revealed by XRD analysis [33]. The similar phase composition, excepting borocementite, was reported in the case of low alloyed steel EN25 [34] and carbon steels C20, C45, C90 having different carbon concentration [35]. For EN25 steel, the high hardness (1170–1315 HV0.5) borided layer of eutectic microstructure was obtained by laser boriding technique. The effect of laser boronising on maraging steels has not been adequately studied yet. Even though the works mentioned above report a visible hardening effect for different steels, it is known that the boronizing of steels is greatly influenced by the alloying elements present [36]. For example, in the case of carbon steel, a significant impact of carbon concentration on the hardness of borided layers was demonstrated in [35]. Various alloying elements have a different impact on the hardness of borides: titanium, vanadium, chromium and molybdenum increase it, while nickel, copper and aluminum decrease the hardness of FeB by 2000–3000 MPa [37]. Maraging steels have a complex alloying system and, to the best of authors’ knowledge, there are no data on the laser boronising of maraging steel available at the moment.

In the present work, the effect of surface laser boronizing on the microstructure and hardness of maraging steel parts manufactured by LPBF is studied with the aim to evaluate the prospects of improving wear resistance.

## 2. Materials and Methods

### 2.1. Preparations of LPBF Samples

**Figure 1:**
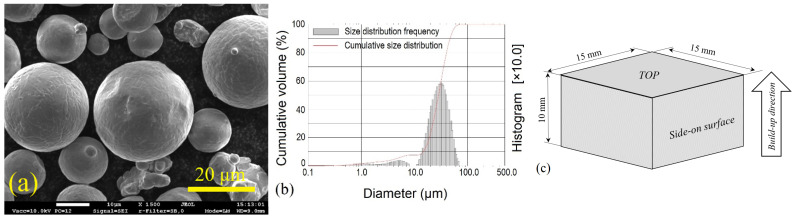
Morphology (**a**) and size distribution (**b**) of powder particles used for the production of samples, and sample drawing (**c**).

The samples for experiments were produced for DIN 1.2709 steel powder (chemical composition in wt.%: 0.03% C; <0.1% Si; <0.1% Mn; (17–19)% Ni; 4.8% Mo; <0.8% Ti; (8.5–9.5)% Co; <0.1% Al; Fe–balance), also known as 18Ni-300 maraging steel. This is one of the most widely used and commercially available maraging steel grades. The powder particle size varied between 7 and 30 μm (as declared by the powder manufacturer). The particles’ morphology observed with a scanning electron microscope (SEM) is shown in Figure 1a. The real particles’ size and its distribution assessed by particle size analyzer Cilas 1090 is given in Figure 1b. For the production of samples by LPBF process, Concept Laser M3 equipment was used. The main characteristics of the equipment and process parameters are listed in Table 1. The square prism specimens with dimensions of 15 × 15 × 10 mm were produced (Figure 1c). The produced samples were separated from the substrate (made of the same steel grade) by electric discharge cutting method using the wire spark erosion machine Charmille Cut 200. The separated samples were cleaned in an ultrasonic bath in C_3_H_8_O solution for 15 min at 40 °C temperature. Seven prism samples were produced, five of which were for the laser boronizing experiments (two of which were for the further aging experiments) and two for the laser processing without alloying additive.

### 2.2. Details of Laser Boronising Process

**Figure 2:**
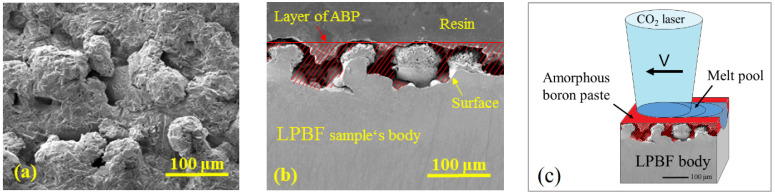
Morphology of side-on surface of as-manufactured LPBF sample (**a**) and a cross-sectional micrograph of LPBF sample with the schema of pre-placing amorphous boron paste (ABP) (**b**); (**c**)–schematic diagram of laser boronizing process.

CO_2_ continuous laser at 1 kW power was used to process the surface of the LPBF samples. Single laser-processed tracks were formed on the side-on surfaces of LPBF samples with amorphous boron paste preplaced on the surface (Figure 2). For the preparation of the paste, amorphous boron powder was mixed with 3 wt.% of organic binder and then with alcohol to obtain fluid consistence. The natural surface roughness (*Ra* = 9.2 μm) and morphology obtained after LPBF process (Figure 2a) were used to control the thickness of the preplaced boron layer (Figure 2b). Laser surface processing was performed by the varying laser spot diameter from 0.5 to 4.0 mm and laser operating speed between 250 mm/min and 1500 mm/min (Table 2). Two main parameters predetermine the process mode, namely the intensity of laser irradiation energy and the duration of its action. Taking into account that the efficiency of the applied laser is ~10%, the laser beam power was ~100 W. The power density (*P*/*d*, W∙m^−2^) can be calculated using laser power *P* and laser beam spot diameter *d*. Laser beam power *P* and operating speed *v* determine the heat input (*P*/*v*, J∙m^−1^). A radiant exposure (J∙m^−2^) that considers both the intensity of the laser power and its action time can by calculated by dividing a heat input by a laser spot diameter. The applied power density, heat input, and radiance exposure values are listed in Table 2. The specimens processed by laser at the same parameters without preplacing amorphous boron were used as a reference. MSt acquire their excellent mechanical properties after appropriate heat treatment. To evaluate the effect of such heat treatment on the boronized surfaces, the aging was applied for the samples and their properties before and after aging were compared. Heat treatment included solution annealing at 840 ± 5 °C for 2 h, cooling in air, and aging at 490 ± 5 °C for 2 h.

### 2.3. Surface Characterization Methods

XPS characterization of laser-modified surfaces was carried out using Kratos AXIS Supra+ spectrometer with monochromatic Al Kα (1486.6eV) X-ray radiation powered at 225 W. The base pressure in the analysis chamber was less than 1 × 10^−8^ mbar and a low electron flood gun was used as charge neutralizer. The survey spectra for each sample were recorded at pass energy of 40 eV in 0.5 eV step and high-resolution spectra (pass energy 10 eV, in 0.1 eV steps) over individual element peaks. The binding energy scale was calibrated by setting the C1s hydrocarbon peak at 284.8 eV. For sputter experiments the monoatomic Ar+ ion gun (Minibeam 6) with an energy of 5 keV and 110 µm aperture was used. The effective sputtering rates were 3.2 nm/min measured at a SiO_2_ reference sample. After sputtering for 90 s, high resolution scan spectrum for each element was obtained and the chemical composition was determined from corresponding XPS peak areas after inelastic background subtraction and using the relative sensitivity factor method (sensitivity factors are included in the ESCApe software, Kratos).

Phase composition of the modified surface was studied by X-ray diffraction (XRD) analysis. XRD patterns were measured using an X-ray diffractometer SmartLab (Rigaku) equipped with an X-ray tube with a 9 kW rotating Cu anode. The measurements were performed using Polycapillary Focusing Optics with Bragg–Brentano geometry and a graphite monochromator on the diffracted beam. Step scan size was of 0.02° (in 2θ scale) and counting time of 1 s per step. The measurements were conducted in the 2θ range of 10–75°. Phase identification was performed using the software package PDXL (Rigaku) and the ICDD powder diffraction database PDF4+ (2019 release).

The microstructural analysis was carried out on polished and etched cross-sections using a JEOL JSM-7600F scanning electron microscope (SEM) coupled with an energy dispersive spectrometer (EDS) for X-ray microanalysis. The last polishing step was carried out using fumed silica polishing suspension with a grit size of up to 0.2 µm. For the etching, the mixture of H_2_O, CH_3_COOH, HCl and HNO_3_ in ratio 1:1:4:1 was used; etching time was 30 s. The SEM/EDS analysis parameters used: 10 kV accelerating voltage; imaging with 50% backscattered and 50% secondary electrons; 8 mm working distance. Moreover, the coloring etching method was applied to reveal the presence of borides. The polished micro-sections were etched by sodium picrate etchant for 30 s (37 ± 1 °C) and analyzed with the optical microscope. Typically, sodium picrate colors FeB borides in blue and Fe_2_B borides in brown. The geometry of the melt pools obtained at different laser processing parameters was analyzed from the cross-sectional SEM micrographs. The depth and the width of the melt pools were measured using SEM JEOL JSM-7600F software for image analyzing and measurements. An area of melt pool was calculated from the micrographs of melt pools applying the Scion Image software for image analysis. The presented values of the area, depth and width were calculated as a mean of three measurements obtained on three different cross-sections.

Microhardness measurements were carried out by Knoop method using a ZwickRoell ZHμ tester. The measurements were performed on polished cross-sections. For depth hardness profiles, the microhardness was measured upon a formation of a sequence of dents with a 25 μm step in the direction of the processed surface thickness with a 0.05 kg load and 10 s indentation duration. The profiles presented were created based on the individual measured values. The hardness average was calculated as a mean of nine measurements (three indentations on three cross-sections) made with a 0.2 kg load and 15 s indentation duration. The average hardness is presented with a standard deviation calculated as a STDEV.P function using Microsoft Excel software.

## 3. Results

### 3.1. Geometry of Laser Processed Passes

**Figure 3:**
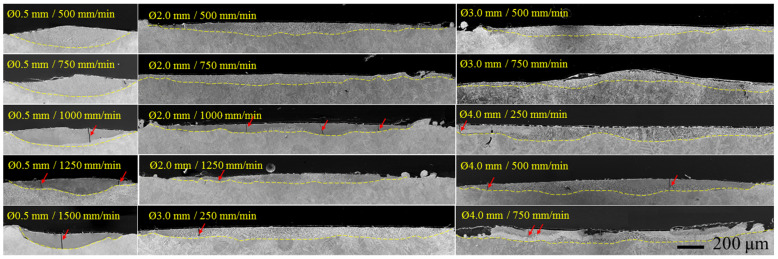
General view of the melt pools obtained by laser boronizing at different parameters; red arrows show the location of transverse cracks.

The micrographs of the re-melted zone cross sections of the specimens laser-processed with boron addition are shown in Figure 3. In the literature, the ~10^3^–10^5^ W∙cm^−2^ power density interval is indicated as typical for the metal melting mode. Here, the melting of the surface occurred within all the applied intervals of power density and radiance exposure; however, the processing at 2.0, 3.0 and 4.0 mm laser spot diameters, which provided power density up to 3184 W∙cm^−2^ and radiance exposure up to 8.0 J∙mm^−2^, allowed neither a stable pool width nor thickness to be obtained (Figure 3). The most acceptable pool geometry with sufficient melting depth was obtained at 0.5 mm laser spot diameter, providing the highest power density, 5.1∙10^4^ W∙cm^−2^, from the chosen range and laser radiance exposure between 8 and 24 J∙mm^−2^. Taking into account these obtained results, the more detailed analysis was performed only for layers laser-boronized at 0.5 mm diameter. The geometry of laser pools obtained with boron did not differ visibly from those obtained without boron (the micrographs are not presented in the paper). At the same time, the transvers cracks were observed in the samples processed with boron. Transverse cracks are a known problem of thermochemically borided layers. During laser boronizing, the unmelted metal surrounding the melt pool in the specimen restrains free shrinkage of the melted surface metal and dilatation of heated material’s layers. As a result, thermal tensile stresses arise, the level of which is largely predetermined by the chemical composition and properties of the melt/alloy. Maraging steels are not sensitive to thermal stresses. However, the addition of boron to steel changes its chemical composition and physical and mechanical properties. Moreover, the plasticity of borides, which are formed with the boron addition, is negligibly small; respectively, the plasticity of the surface layer decreases after boronizing. The insufficient resistance to tensile stresses of both the melt and crystallized layer containing brittle borides may be the reason for the occurrence of transverse cracks here.

**Figure 4:**
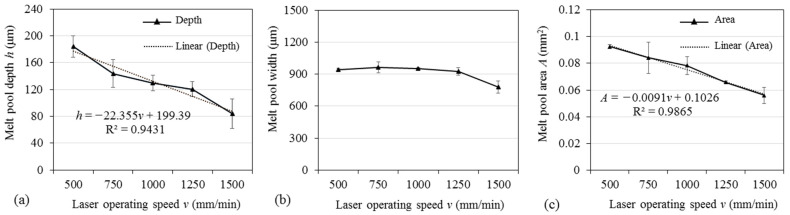
Depth (**a**), width (**b**) and area (**c**) of melt pool obtained by laser processing at 0.5 mm laser spot diameter with boron in relation to laser operating speed.

For the laser alloying process, the geometry of the melt pool and its controllability are of high importance since it has a direct impact on the mixing proportion between the alloying element and the parent metal, and, respectively, on the concentration of the alloying element (boron in this case) in the alloyed layer and its properties. At 2.0, 3.0, and 4.0 mm laser spot diameter, the depth and width of the melt pool visibly varied and no clear dependency on the processing parameters was observed. At 0.5 mm laser spot diameter, the maximum melting depth varied from ~84 μm to 184 μm and was in a strong dependency on the applied laser operating speed (Figure 4a), which can be described by linear regression with sufficient accuracy (R^2^ = 0.9431). The melt pool width did not change visibly in the laser speed range from 500 to 1250 mm/min with typical width values of ~920–960 μm. The decrease in melt pool width was determined when the laser operating speed reached 1500 mm/min. The area of melt pool cross-section ranged from ~5.6∙10^−2^ mm^2^ to ~9.2∙10^−2^ mm^2^ and was dependent on the laser operating speed as well. A noticeable variation in depth, width and, respectively, area was determined within the passes. Most likely, the primary surface morphology obtained after the LPBF process was not even enough to provide a stable laser focal distance. Moreover, the unevenness in the primary surface morphology and flatness could result in a local variation in the pre-placed boron paste layer thickness, which had an additional effect on laser energy absorption, dissipation and distribution in the surface layers. As a result, the depth of the melt pool varied visibly and its width varied in a less degree. Taking into account the results of primary evaluation of the obtained laser-processed tracks, the more detailed further analysis was performed only for the layers that were laser-boronized at 0.5 mm laser spot diameter.

### 3.2. XPS Characterization of Processed Layers

The elemental composition of laser-boronized surfaces according to XPS analysis is given in Table 3. The presence of boron was established for all laser-alloyed samples. The concentration of boron varied between ~3.1 wt.% and ~5.7 wt.% and showed a strong dependency on laser operating speed that predetermines the melt pool area and, respectively, the boron and parent metal mixing ratio during the melting process. Besides boron and all main components of the parent metal (Fe, Ni, Co, Mo), XPS revealed a significant concentration of carbon and oxygen. The specimens were pre-polished to remove ~15 μm of the surface layer before XPS analysis; the XPS spectrum was recorded after surface sputtering with monoatomic Ar+ ion gun for 90 s. Possibly, the applied sputtering time was not enough to fully remove the carbon and oxygen that are inevitably adsorbed on the surface from the atmosphere. Boron may act as a de-oxidizer as well [38]. LPBF process was performed in Ar shielding gas; however, the surface of LPBF part consists of metal oxides due to further natural oxidation in air. During the reaction of boron with metal oxides, boron oxides containing compounds may be formed, which due to low density are concentrated in the top part of the melt pool. This may also be the reason for the increased oxygen concentration. The EDS analysis performed on the cross-sections of specimens did not show a high oxygen concentration (1.2–1.9 wt.% O by EDS, see cl. 3.4).

**Figure 5:**
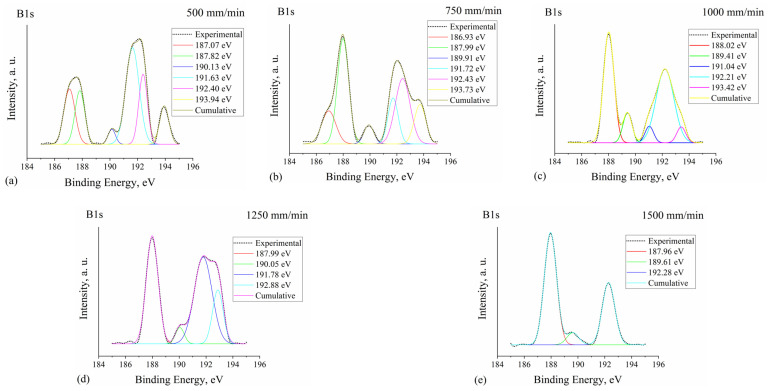
XPS spectra for B1s peaks with fitting curves for the specimens laser boronized at 500 mm/min (**a**), 750 mm/min (**b**), 1000 mm/min (**c**), 1250 mm/min (**d**) and 1500 mm/min (**e**) laser operating speeds.

Figure 5a–e shows XPS spectra B1s for the specimens laser-boronized at different laser operating speeds and containing different boron concentration. Deconvolution of B1s spectra of the specimen processed at 500 mm/min revealed several peaks between ~187 eV and ~194 eV binding energies (BE) (Figure 5a). The peak at ~187 eV is attributable to elemental boron [39]. For specimen “500 mm/min” this peak is overlapped with another one at 187.8 eV of similar intensity. Based on the literature [39,40], the B1s peaks between ~187.3 and ~188.4 eV may be attributed to metal borides including FeB and Fe_2_B. When the laser speed was increased to 750 mm/min, and the boron concentration to ~4.1 wt.%, the intensity ratio of these two peaks changed in favor of the peak at a higher BE that corresponds to borides. With a further increase in laser speed and boron concentration, the peak at ~187 eV disappeared and only a single peak at ~188 eV was observed, which in works dealing with steel processing is associated mainly with Fe_2_B borides [41,42]. The B1s peaks between ~191 eV and ~194 eV are mainly associated with boron oxides [39,40,42,43]. In [43], a broad B1s peak between ~190 eV and ~194 eV was considered to be due to the formation of non-stoichiometric oxides; three equally spaced subpeaks were attributed to B–O (lowest BE), O–B–O and B_2_O_3_ (highest BE).

In the present study, three similar peaks (~191.0–191.7 eV; ~192.2–192.4 eV; ~193.4–193.9 eV) were observed after deconvolution of B1s spectra for the specimens processed at 500, 750 and 1000 mm/min laser speed; at 1250 mm/min laser speed one of peaks disappeared; at 1500 mm/min only one was left (~192.3 eV). The presence of oxides in this case has only surface nature and does not influence the properties of the modified layer in general; therefore, these peaks are not of primary importance in this study. At the same time, one more peak at 189.4–190.1 eV was observed in all the studied specimens. XPS handbook [39] attributes B1s peaks between 189.8 eV and 190.2 eV to a BN compound, which here may be the result of an amorphous boron reaction with nitrogen from air at a high temperature during laser processing of the surface. In another source [44], the B1s peak at 189.9 eV is attributed to Ni_2_B boride that here could be formed in laser-modified layers as well.

**Figure 6:**
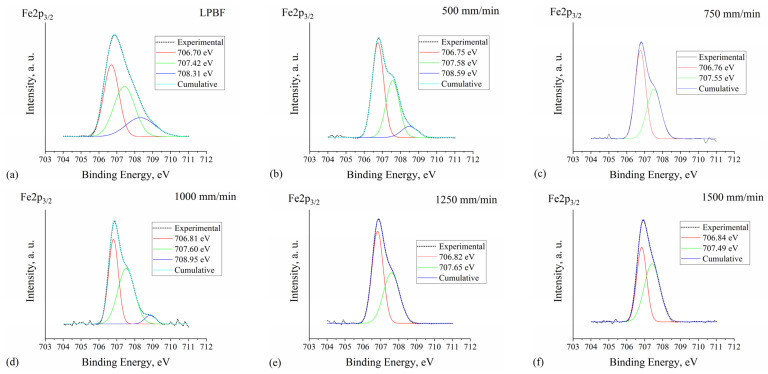
XPS spectra for Fe2p2/3 peaks with fitting curves for the LPBF specimen (**a**) and specimens laser boronized at 500 mm/min (**b**), 750 mm/min (**c**), 1000 mm/min (**d**), 1250 mm/min (**e**) and 1500 mm/min (**f**) laser operating speeds.

Figure 6a–f shows XPS spectra Fe2p3/2. After the deconvolution, two peaks at 706.70–706.84 eV and 707.42–707According to the XPS handbook [39], the Fe2p3/2 peak at 706.75 eV corresponds to metallic Fe (Fe^0^); for iron borides this peak has only a slight shift of ~0.1 eV–to lower energy for Fe_2_B and to higher energy for FeB. Here, the tendency of a slight Fe^0^ peak shift to higher binding energies was observed with the addition of boron and with the increase in boron concentration: 706.70 eV (no boron, LPBF) → 706.75 eV (3.14 wt.% B) → 706.76 eV (4.05 wt.% B) → 706.81 eV (4.96 wt.% B) → 706.82 eV (5.37 wt.% B) → 706.84 eV (5.73 wt.% B). The shift is very small, ranges between ~0.05 and 0.15 eV and is similar to that indicated for iron borides. However, a gradual increase in this shift with an increase in boron concentration shows that it may also be due to the formation of a boron solid solution in iron. The solubility of boron in iron is limited and very small; however, unlike carbon, boron forms substitutional solid solutions with iron [45,46]. The electron BE, for an atom in the alloy, may differ when compared with pure metal due to the change in atomic local environments [47]. According to [40,48], the Fe2p3/2 peak at 707.42–707.65 eV, which was observed for all the specimens, and the peak at 708.31–708.95 eV, which appeared for specimens “LPBF”, “500 mm/min” and “1000 mm/min”, may highly likely be attributed to the non-stoichiometric oxides Fe∙Ox; apparently, the stoichiometry of the last one may be close to that of FeO oxide, for which ~709.2 eV BE is given in [39].

### 3.3. X-ray Diffraction Analysis

**Figure 7:**
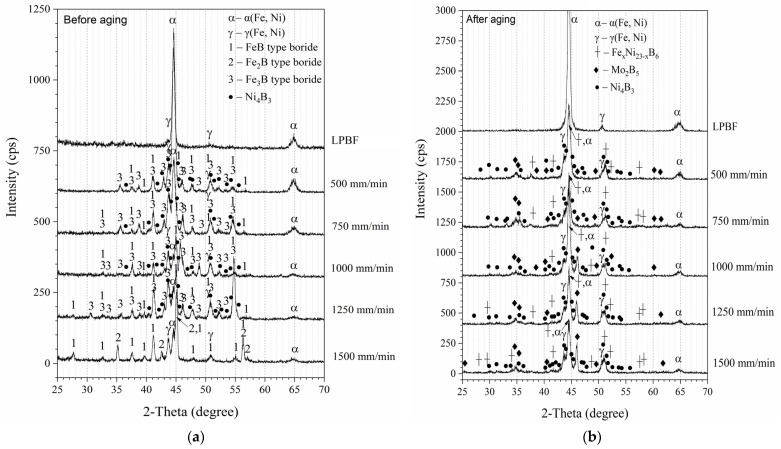
XRD patterns of as-manufactured LPBF specimen (LPBF) and specimens boronized at different laser operating speeds (from 500 mm/min to 1500 mm/min) before (**a**) and after (**b**) aging.

XRD patterns for the as-manufactured LPBF specimen (LPBF) and specimens laser-boronized at a 0.5 mm laser spot diameter and different laser operating speed are presented in Figure 7a. The major reflections observed in the XRD pattern of the LPBF specimen may be attributed to a α (Fe, Ni) type phase, having cubic crystal lattice with parameter a = 2.8681 Å. Taking into account the high cooling rates during the LPBF process, this phase can be identified as a martensitic phase that is typical for low carbon maraging steels [49]. The minor reflections may be attributed to a phase such as γ (Fe, Ni) austenite, having a = 3.5975 Å. With the addition of boron, the reflections attributable to boride phases appeared in diffraction curves. Due to the complex alloying system of maraging steel, it is difficult to establish unambiguously a phase composition as the reflections observed in XRD patterns may be attributed to a number of different borides. Low intensity and absence of some peaks makes it difficult as well. The positions (2-Theta angle) of the observed peaks were in good consistence with the data given in the XRD database for such phases as FeB, Fe_2_B, and Fe_3_B borides. Alloying elements (Mo, Co, Al, Ti) may replace iron in both solid solution and boride phases or form other minor phases, the amount of which is not enough to form intensive reflection in the XRD pattern. In the specimens that were laser-boronized at lower laser speeds (500–1000 mm/min) and contained a lower amount of boron (~3–5 wt.%), the presence of reflections attributable to a lower Fe_3_B type boride was established along with the presence of low-expressed peaks that may belong to FeB type boride. With further increase in laser speed to 1250 mm/min and boron content to ~5.4 wt.%, more intensive peaks attributable to FeB were observed, indicating the increase in that type boride amount.

In the specimens that were laser-boronized at 500–1250 mm/min laser speeds, the low-intensity peaks at 2-theta between 36° and 37° and between 40° and 41° were observed, which cannot belong to the above-mentioned borides; however, taking into account the possible overlapping with other reflections, they may be attributed to Ni_4_B_3_ phase with high certainty. Finally, at 1500 mm/min, the reflections typical for Fe_3_B boride almost disappeared and those attributable to Fe_2_B type boride appeared, while FeB phase remained and a slight increase in FeB peak intensity was observed. At the same time, a slight decrease in intensity of α(Fe, Ni) peaks at 2-theta ~64.98° can be seen with the increasing laser operating speed. The results of the XRD analysis for non-aged samples, in general, are consistent with XPS results, the results are reported on the metastable Fe-B phase diagram obtained at undercooling conditions in [31], and with the data presented in the above-mentioned works on laser boriding of steels [32,33,34,35]. The XRD pattern of the LPBF sample, after aging, did not differ from that of a non-aged sample (Figure 7b). For laser-boronized layers, the view of XRD patterns changed after aging. Besides the reflections attributable to martensite and austenite phases, the reflections, that with high probability may belong to Mo_2_B_5_ and Fe_x_Ni_23-x_B_6_ compounds, were identified. Ni_4_B_3_ phase was identified after aging as well.

### 3.4. Microstructural Analysis

**Figure 8:**
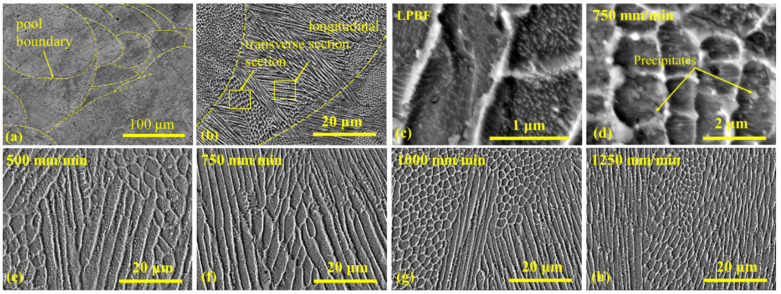
SEM images of LPBF body microstructure (**a**–**c**) and microstructure of laser processed surfaces without alloying additive (**d**–**h**): (**a**) overview showing substructure of LPBF specimen due to “point-by-point” building pattern; (**b**) longitudinal and transverse directions of cellular microstructure of LPBF body; cellular microstructure of LPBF at high magnification (**c**); cellular microstructure and dispersive precipitations in the surface processed without boron at 750 mm/min laser speed (**d**); cellular microstructure of laser processed surfaces at 500, 750, 1000 and 1250 mm/min laser speed, respectively (**e**–**h**).

The microstructure of additively manufactured maraging steel parts (Figure 8a–c) differs from that of parts conventionally manufactured. The LPBF presents successive processes of steel powder melting by laser, when the 3D part is built-up “layer-by-layer” and “point-by-point” within each layer. As a result, a substructure is formed, that consists of individually solidified micro-volumes with their own boundaries (Figure 8a). Inside each micro-volume, the fine submicron-sized cellular microstructure (Figure 8b) is observed that is typical for maraging steel parts obtained through the LPBF process [8,9,10,11,12,13,14]. A similar microstructure was formed after the laser processing of the LPBF part surface without boron addition, (Figure 8d–h); however, after laser processing, a precipitation of submicron-sized crystals was observed (Figure 8d), indicating that a partial aging effect might take place when already processed layers were heated during laser processing of the neighboring surface areas. It should be also pointed out that the microstructure after the laser-processing of the surface was slightly coarser compared to after the LPBF process, which is due to there being less concentrated heating during the surface processing as compared with the build-up process (Figure 8e–h). At the same time, the transition from coarser to finer morphology with an increased laser speed can be seen clearly.

**Figure 9:**
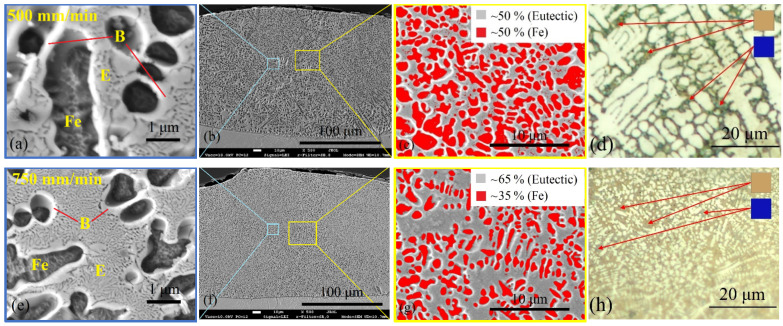
Hypoeutectic microstructure of the layers laser-boronized at 500 (**a**–**d**) and 750 (**e**–**h**) mm/min laser operating speed; (**a**,**b**): Fe-based solid solution dendrites (Fe) surrounded by the boride shell (B) that separates them from the eutectic phase (E); (**b**,**f**)–general view of the melt pool with denoted areas of images (**a**,**c**,**e**,**g**); (**c**,**g**)–micrographs processed with Scion Image program to assess the volume ratio of eutectic and solid solution phases; (**d**,**h**)–optical micrographs of microstructure after etching with sodium picrate, which typically colors FeB borides in blue and Fe_2_B borides in brown.

**Figure 10:**
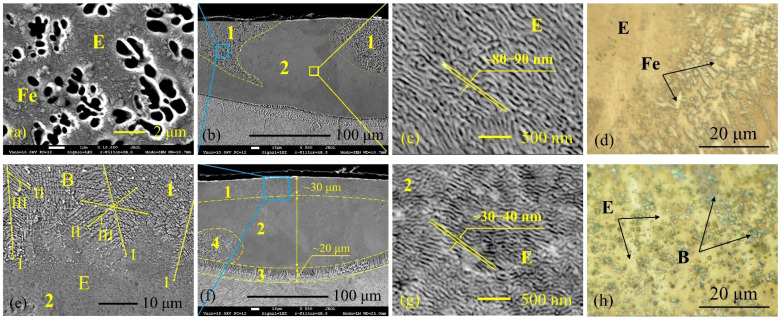
Near-eutectic microstructure of the layers laser-boronized at 1000 (**a**–**d**) and 1250 (**e**–**h**) mm/min laser operating speed; (**a**) area of hypoeutectic structure—Fe-based solid solution dendrites (Fe) surrounded by the boride shell, which separates them from eutectic phase; (**b**) a general view of the melt pool—mainly eutectic structure (2) with areas of hypoeutectic (1); (**c**) and (**g**)—lamellar nanoeutectic structure under higher magnification; (**d**) and (**h**)—optical micrographs of microstructure after coloring etching; (**e**) top of melt pool—boride dendrites (arrows show direction of primary (I), secondary (II) and third (III) dendrites’ arms); (**f**) general view of melt pool with denoted four zones; E—eutectic; Fe—γFe-based solid solution; B—boride; (3)—a layer with Fe-based solid solution dendrites; (4)—area of hypoeutectic structure.

**Figure 11:**
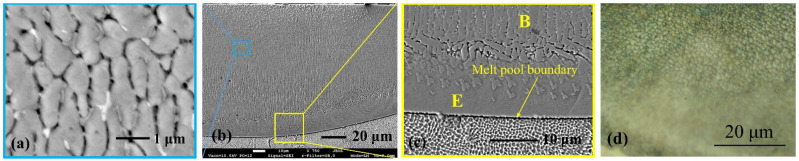
Hypereutectic microstructure of layer laser-boronized at 1500 mm/min laser operating speed: (**a**) structure of ultra-fine dendritic borides and residues of eutectic between them; (**b**) general view of the melt pool—mainly dendritic microstructure with dendrite direction variation in various melt pool areas; (**c**) layer of planar solidification near melt pool boundary with boride dendrites in transvers direction; (**d**) optical micrograph of microstructure after coloring etching; E—eutectic; B—borides.

The addition of boron changed the microstructure significantly. The evolution of the microstructure, with the increase in boron concentration from ~3.1 wt.% to ~5.7 wt.%, is shown in Figure 9, Figure 10 and Figure 11. At ~3.1 wt.% B and ~4.1 wt.% B, the microstructure showed typical hypoeutectic solidification of primary γ-Fe phase and very fine lamellar eutectics of boride-based eutectic (Figure 9a,e). It can be seen as well that iron solid solution dendrites are surrounded by boride shell, which separates them from eutectic regions. Such microstructure has formed throughout the entire melt pool volume (Figure 9b,f). With the increase in boron concentration from ~3.1 wt.% B up to ~4.1 wt.% B, the ~15% increase in eutectic phase was established (Figure 9c,g). The microstructure also became finer and that is related with the increased laser operating speed and, respectively, heating/cooling rates. For microstructural analysis, the coloring etching of the samples was additionally performed using sodium picrate solution, which typically colors FeB borides in blue and Fe_2_B borides in brown. As can be seen in Figure 9d,h, the areas between white dendrites (γ-Fe phase) are brown-colored. The literature sources do not give any information about the reaction of sodium picrate solution with other types of borides (besides FeB and Fe_2_B). Taking into account the results of XRD analysis, one may assume that Fe_3_B type boride phase was colored in brown here and brown-colored areas are Fe_3_B-type-borides-based eutectic. Very fine blue points can be seen around white dendrites as well (Figure 9d,h). Presumably, FeB-type boride shell surrounds primary dendrites of γ-Fe solid solution. The EDS analysis results for eutectic and solid solution regions are presented in Table 4. Boron and carbon were eliminated from the results, since the sensitivity of EDS to low-weight elements is poor and the inclusion of such elements leads to a significant distortion of quantitative results. It should be noted that when boron was included, EDS showed ~3-4 times higher boron concentration in the eutectic region (~9.5 wt.%) as compared with iron-based solid solution areas (~2–3 wt.%). A slightly higher concentration of iron (by ~2 wt.%) and nickel (by ~3 wt.%) was established for the iron-based solid solution phase (Spectrums 3 and 4), while molybdenum and titanium were mostly concentrated in boride-containing eutectic phase (Spectrums 1 and 2). Map analysis of all the specimens showed a practically even distribution of all the elements, indicating that the difference in element concentration for different phases is insignificant. It can be stated from the results of EDS that FeB, Fe_2_B, and Fe_3_B type borides identified by XRD analysis are very likely to be the complex borides MB, M_2_B and M_3_B, where M = Fe, Ni, Co, Mo, Ti.

When boron concentration ranged from ~5 wt.% to ~5.4 wt.% (specimens laser processed at 1000 and 1250 mm/min laser speed, respectively), the prevailing superfine lamellar nano-eutectic structure was established (zone “2” in Figure 10b,f) with slightly thicker boride lamella (~80–90 nm) obtained at a lower laser speed and thinner boride lamella (~30–40 nm) at a higher laser speed (Figure 10c,g). At ~5 wt.% B, the minor presence of some Fe-based solid solution phase was observed locally, which was dissolved after etching (Figure 10a; zone “1” in Figure 10b). At ~5.4 wt.% B, a ~30 μm thick layer of boride dendrites located in the upper part of the melt pool was observed (Figure 10e; zone “1” in Figure 10f). The prevailing crystallization direction of these dendrites is almost vertical from the top to the melt pool inside. In the bottom of the melt pool, a ~20 μm thick layer of Fe-based solid solution dendrites was formed.

A similar distribution of phases was observed during the microstructural analysis of the layers laser-processed at other laser spot diameter as well, which allows for the assumption that some densimetrical effect may take place, due to which the concentration of lightweight boron is slightly higher at the top of the melt pool and slightly lower at the bottom. The formation of such microstructure gradient may be considered as a beneficial effect when hard phases are distributed in the main working layer, while a leap of properties is smoothed. The sodium picrate colored these samples mainly in brown color (Figure 10d,h).

A further increase in B concentration up to ~5.7 wt.% resulted in a formation of a submicron-sized dendritic boride structure with dendrites across sizes ~0.5–1.0 μm (Figure 11a). The direction of dendrites differed in different zones of the melt pool, which was pre-determined by the dynamically changing melt pool geometry during laser processing (Figure 11b). Near the melt pool boundary, a thin layer of eutectic structure was observed, followed by a layer of boride dendrites (transverse cross-section) solidified along the laser movement direction; furthermore, the transition zone can be seen, where the direction of dendrites has some angle with laser track axes, after which the zone of mainly vertical dendrites begins (Figure 11c). Between boride dendrites, the places of etched out phase can be seen (most likely, Fe-based solid solution) along with white non-etched inclusions (most likely boride of other type than dendritic). A coloring etching resulted mainly in a brown color and very fine blue points (Figure 11d).

Taking into account the results of XRD analysis, one may assume that the hypereutectic microstructure, formed at 1500 mm/min laser speed, mainly consists of M_2_B-type and MB-type borides.

In general, the observed microstructure evolution of laser-boronized layers in the obtained boron concentrations’ range is consistent with Fe-B phase diagram.

### 3.5. Effect of Aging on Microstructure of Laser-Boronized Layers

**Figure 12:**
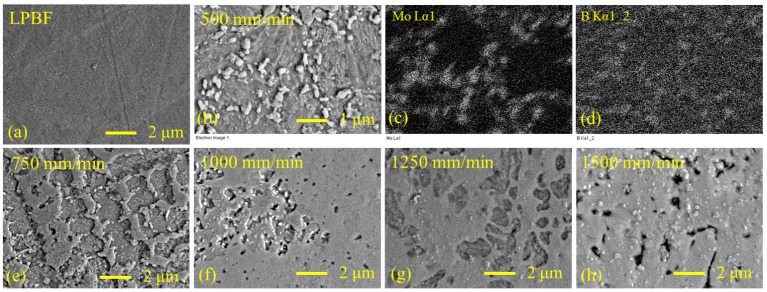
Microstructure of LPBF sample (**a**) and laser-boronized layers (**b**,**e**–**h**) after aging; (**c**) and (**d**) distribution maps of molybdenum and boron for the sample laser-boronized at 500 mm/min laser operating speed; (**b**) and (**e**–**h**) white precipitates rich in molybdenum and boron.

**Figure 13:**
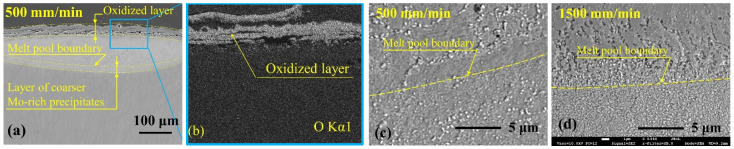
General view of the melt pool after aging (**a**) and oxygen distribution map of the melt pool top (**b**), plenty precipitation of molybdenum rich particles in parent metal near the melt pool boundary (**c**) and (**d**).

Aging of the LPBF part has resulted in a very fine plenty precipitation (Figure 12a). According to the literature sources, Fe_2_Mo are formed during aging. In laser-boronized layers, besides a very fine uniform precipitation within all phases, coarser precipitates were observed (white particles in Figure 12b,e–h). According to EDS mapping, these particles are rich in molybdenum and boron (Figure 12c,d). Taking into account the results of the XRD analysis, these precipitates may be identified as Mo_2_B_5_. These precipitates were mainly observed at the Fe-based solid solution dendrite boundaries (Figure 12b,e,f) and, in a lower extent, within the dendrites and other phases (Figure 12g,h). Aging of samples was performed in an electrical furnace in air. As a result, a ~30–40 μm thick surface layer was oxidized (Figure 13a,b). At the same time, a ~20 μm layer containing Mo- and B-rich precipitates was formed in a parent metal near the melt pool (Figure 13a), indicating that boron diffusion into the parent metal took place. The precipitation is more ample and finer in the samples laser-boronized at a higher laser operating speed (Figure 13c,d), which is related to higher boron concentration and cooling rate.

### 3.6. Hardness of Laser-Boronized Layers

**Figure 14:**
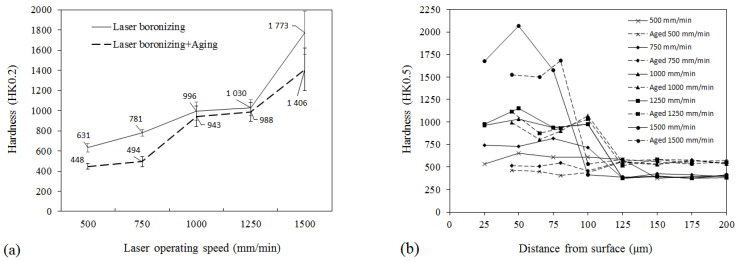
Average hardness of laser-boronized layers (**a**) and hardness depth profiles (**b**) before and after aging.

As was established by the measurements, the hardness of as-manufactured LPBF part is ~380–420 HK0.2. The hardness of the layers laser-processed without boron was between ~390 and ~570 HK0.2 and there was not any expressed correlation with laser processing parameters observed. The highest hardness numbers are comparable with those of maraging steel after full aging. Taking into account the observed precipitation (Figure 8d), the obtained increase in hardness may be associated with the aging processes of LPBF, partly due to the heating of already laser-processed layers during the laser processing of the neighboring layers. The hardness of boronized layers was significantly higher and ranged between ~630 and ~1770 HK0.2 with a strong hardness dependence on the laser operating speed (Figure 14a). For the hypoeutectic microstructure formed at 500 and 750 mm/min laser speeds, the hardness was ~630 and ~780 HK0.2, respectively. The hardness of ultrafine nano-eutectic was ~1000–1030 HK0.2 and this is comparable with the hardness after thermochemical nitriding. For hypereutectic microstructure with fine dendritic borides prevailing, the hardness numbers were close to those of iron borides. Obviously, the increase in hardness with increasing laser speed is mainly predetermined by an increase in boride phase due to the increase in boron concentration associated with the decrease in melt pool area. The hardness additionally increases due to a higher heating/cooling rate caused by a higher laser operating speed, resulting in the finer microstructure, as was observed in the microstructural analysis. It should be also pointed out that the most significant fluctuations of hardness from ~1550 to ~2000 HK0.2 were observed for the samples having the highest hardness. For these samples, the prevalence of borides in the structure and the transition from lower borides to higher were observed. At the same time, due to the fluctuation of the melt pool area, the variation in local boron concentration might result in a change of the volume ratio between the different types of borides formed, the hardness of which may differ by ~3000–4000 MPa [30]. This explains a significant hardness variation observed at the highest boron concentration. After aging, the hardness of LPBF part increased and was ~585 HK0.2, while the hardness of laser-boronized layers decreased. A more significant decrease by ~200–300 hardness numbers can be pointed out for hypoeutectic samples, while the hardness decrease in the samples with eutectic structure was not so significant. For hypereutectic (near-boride) structure, the hardness drop was ~370 hardness numbers; however, the hardness was still very high (~1400 HK0.2).

Hardness depth profiles for the samples before aging showed a rather uniform hardness distribution in the layers with hypoeutectic microstructure, a slight fluctuation in hardness for near-eutectic layers and a leap of hardness in the middle part of the boronized layer with hypereutectic near-boride microstructure, which possibly may be associated with different orientation of boride dendrites in different melt pool areas (Figure 14b). After aging, a more visible change in hardness distribution was observed for the hardest near-boride sample, while for other samples it did not change significantly. Precipitation of Mo- and B-rich particles in the parent metal near the melt pool boundary did not influence visibly the hardness of these zones. In general, laser boronizing enabled an increase in the surface hardness by ~1.5–4.2 times, as compared to as-manufactured LPBF parts, and up to three times as compared to LPBF parts after aging.

Laser boronising experiments performed in the present study showed that boronized layers with a wide range of hardness (according to some individual measurements, up to ~2000 HK, which corresponds to the hardness of upper borides) may be formed by applying this technique for maraging steel. One of the ideas of this work was to perform laser alloying of LPBF parts without any pre-processing of the surface and to use the surface morphology of LPBF part for controlling the thickness of alloying additive paste. The experiments showed that the morphology was not uniform enough to provide a stable result; however, in general, this method could be used if the uniformity of surface morphology is achieved with the last sintering step. The microstructure of laser-boronized layers, including the morphology of formed borides, differs significantly from that of thermochemically borided layers and is more similar to that reported for Fe-B alloys. Boride-containing eutectic is the main structural constituent present in all the obtained alloyed surfaces. It is reported in [50] that besides high yield strength, Fe-5.7 wt.% B alloy possesses very good plasticity (~19.8%), which the authors attributed to the very fine nano-sized eutectic structure. In the present study, the formation of lamellar nanoeutectic phase with a hardness of ~1000 HK0.2 was established as well. High hardness and sufficient plasticity of nanoeutectic phase obtainable in laser-boronized layers means one can expect a monolithic hard layer may be formed and a significant improvement in wear resistance may be achieved by the application of laser boronizing technique for MSt. The important results obtained on Fe-B alloys in [51] may be mentioned as well. It was found in [51] that alloys having ~3–4 wt.% B and near-eutectic structure have better wear resistance than that of alloys with a higher B concentration. The appearance of harder but more brittle borides at a higher boron concentration did not improve the wear resistance—the signs of borides crumbling were observed leading to the general increase in wear. It is also noticeable that after aging the hardness decrease in near-eutectic layers was insignificant and such a sequence of operations—laser boronising followed by aging—can be applied if the oxidation problem will be solved (for example, performing the aging in inert gas atmosphere). Summarizing the results of the present work and the studies of other researchers, one may assume that, perhaps, it is not purposeful to reach the highest hardness of the alloyed layer. A certain balance between hardness and plasticity should be found that would provide good performance of alloyed surface.

## 4. Conclusions

In the present study, the laser boronizing technique was applied to modify the surface of 18Ni-300 maraging steel parts manufactured by selective laser melting and to increase its hardness. The following conclusions were drawn from the results of experimental research described in the present paper:

1. When surface morphology formed after the LPBF process was used to control the thickness of pre-placed amorphous boron paste, surface melting with continuous CO_2_ laser at 50,955 W∙cm^−2^ power density and heat input between 24.0 and 4.0 J∙mm^−1^ provided boron concentration in the alloyed layer between ~3.1 and ~5.7 wt.%. Boron concentration was strongly dependent on the heat input; the growth of boron concentration with the laser operating speed was predetermined by the gradual decrease in the melt pool area.
2. For the layers laser-boronized at 500–1250 mm/min laser speed and containing ~3.1–5.4 wt.% B, XRD analysis revealed the domination of Fe_3_B-type borides along with the presence of FeB-type borides and the presence of reflections attributable to austenitic and martensitic phases. At 5.7 wt.% B, obtained at 1500 mm/min laser speed, Fe_2_B-type borides replaced Fe_3_B-type borides. According to EDS analysis, the borides formed after laser boronizing. Moreover, iron also contain nickel, cobalt, molybdenum and titanium.
3. The microstructure of laser-boronized layers was changing with an increasing boron concentration as follows: hypoeutectic, consisting of fine γ-Fe dendrites surrounded by boride shell, separating them from boride-based eutectic, at ~3.1–4.1 wt.% B; superfine boride-based lamellar nano-eutectic structure (at ~5.0–5.4 wt.% B) with lamella thickness ~80–90 nm at 1000 mm/min laser speed and lamella thickness ~30–40 nm at 1250 mm/min laser speed; hypereutectic, consisting mainly of submicron-sized dendritic boride, at ~5.7 wt.% B.
4. The formation of boride-based microstructure in laser-boronized layers provided the hardness from ~630 to ~1770 HK0.2, which was in a strong dependence on laser speed as well. The obtained hardness was up to three times higher than that of MSt after aging (~585 HK), indicating that laser boronizing technique may be promising in terms of the improvement of MSt wear resistance.
5. The obtained boronized layers showed the tendency for thermal cracking at the applied laser alloying parameters, which was predetermined by their chemical composition and physical and mechanical properties. The presence of significant transverse cracks may worsen the performance of the alloyed surface or make it unsuitable for exploitation. Therefore, for the further development of this technique, it is reasonable to consider the additional means that enable the reduction in or prevention of the formation of cracks.
6. Aging of laser-boronized layers results in a change in phase composition and microstructure, which is mainly expressed in the ample precipitation of Mo_2_B_5_ borides and lead to the reduction in hardness—more significant (by ~200–300 HK0.2) for hypoeutectic and hypereutectic layers and insignificant (by ~50 HK0.2) for near-eutectic layers.

## Figures and Tables

**Table 1:** Characteristics of LPBF process parameters.

Laser Wave Length (nm)	Laser Power, (W)	Laser Spot Size (mm)	Thickness of Single Layer (mm)	Sintering Rate (m∙s^−1^)	Shielding Gas Type	Shielding Gas Consumption (L∙h^−1^)
1064	100	0.2	0.03	0.2	Ar	0.75

**Table 2:** Applied laser radiance exposure (in J∙mm^−2^) at 0.5–4.0 mm laser spot diameter and 250–1500 mm/min laser operating speed.

Laser Spot Diameter (mm)/Applied Power Density (W∙cm^−2^)	Laser Operating Speed (mm∙min^−1^)/ Heat Input (J∙mm^−1^)					
	250/24.0	500/12.0	750/8.0	1000/6.0	1250/4.8	1500/4.0
0.5/50955	-	24	16	12	9.6	8
2.0/3184	-	6	4	3	2.4	2
3.0/1415	8	4	2.7	-	-	-
4.0/796	6	3	2	-	-	-

Experiment was not performed.

**Table 3:** Elemental composition of laser-alloyed surfaces (by XPS; after Ar sputtering for 90 s).

Laser Operating Speed (mm∙min^−1^)	Element Concentration (wt.%)							
	B	C	O	Mo	Fe	Co	Ni	Total
500	3.14	5.25	13.32	7.54	42.47	10.77	17.52	100
750	4.05	5.83	15.24	6.58	46.70	9.10	12.49	100
1000	4.96	5.06	15.29	4.11	47.10	10.52	12.97	100
1250	5.37	6.72	15.33	4.79	44.08	9.82	13.89	100
1500	5.73	4.72	15.96	4.92	44.98	9.83	13.86	100

**Table 4:** Elemental composition of Fe-based solid solution and boride-based eutectic phase in hypoeutectic laser-boronized layers (by EDS; in wt.%).

Element	Eutectic		Solid Solution		EDS Point Places (Sample: 750 mm/min Laser Speed)
	Spectrum 1	Spectrum 2	Spectrum 3	Spectrum 4	
O	1.87	1.82	1.23	1.39	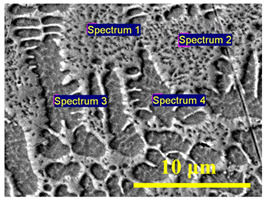
Al	0.01	0.02	0.02	0.02	
Si	0.06	0.08	0.05	0.08	
Ti	0.22	0.24	0.00	0.08	
Fe	59.37	59.18	61.09	61.17	
Co	10.57	11.06	9.76	10.02	
Ni	23.61	23.35	26.17	25.77	
Mo	4.29	4.26	1.67	1.44	
Total	100	100	100	100	

## Data Availability

Not applicable.
